# Environmental surveillance identifies multiple introductions of MRSA CC398 in an Equine Veterinary Hospital in the UK, 2011–2016

**DOI:** 10.1038/s41598-017-05559-8

**Published:** 2017-07-14

**Authors:** Alessio Bortolami, Nicola J. Williams, Catherine M. McGowan, Padraig G. Kelly, Debra C. Archer, Michela Corrò, Gina Pinchbeck, Christine J. Saunders, Dorina Timofte

**Affiliations:** 1 Institute of Veterinary Science, University of Liverpool, Neston, Wirral CH64 7TE UK; 2 Institute of Infection and Global Health, University of Liverpool, Neston, Wirral CH64 7TE UK; 3 Institute of Ageing and Chronic Disease, Health and Life Sciences, University of Liverpool, Neston, Wirral CH64 7TE UK; 4 Istituto Zooprofilattico Sperimentale delle Venezie, Legnaro, 35020 Italy

## Abstract

Bacterial environmental and surgical site infection (SSI) surveillance was implemented from 2011–2016 in a UK Equine Referral Veterinary Hospital and identified 81 methicillin-resistant *Staphylococcus aureus* (MRSA) isolates. A cluster of MRSA SSIs occurred in early 2016 with the isolates confirmed as ST398 by multilocus sequence typing (MLST), which prompted retrospective analysis of all MRSA isolates obtained from the environment (n = 62), SSIs (n = 13) and hand plates (n = 6) in the past five years. Sixty five of these isolates were typed to CC398 and a selection of these (n = 38) were further characterised for resistance and virulence genes, SCC*mec* and *spa* typing. Overall, MRSA was identified in 62/540 (11.5%) of environmental samples, 6/81 of the hand-plates (7.4%) and 13/208 of the SSIs (6.3%). *spa* t011 was the most frequent (24/38) and Based Upon Repeat Pattern (BURP) analysis identified *spa* t011 as one of the two group founders of the main *spa* CC identified across the five years (*spa* CC011/3423). However, 3 singletons (t073, t786, t064) were also identified suggesting separate introductions into the hospital environment. This long-term MRSA surveillance study revealed multiple introductions of MRSA CC398 in a UK Equine Hospital, identifying an emerging zoonotic pathogen so far only sporadically recorded in the UK.

## Introduction

Nosocomial infections are a major problem in human hospitals^1, 2^ yet they have not been recognised as a potential problem until recently in equine veterinary hospitals. However, developments of hospitalisation facilities in veterinary settings have led to opportunities for transmission of nosocomial pathogens similar to those from human hospitals. Veterinary nosocomial outbreaks with methicillin-resistant *Staphylococcus aureus* (MRSA) were reported as early as 1999 in the United States^3^, followed by reports in Austria^4, 5^, Netherlands^6^, Sweden^7^, Israel^8^ and Japan^9^. In addition, hospital spread and/or infections with other well-known agents of nosocomial infections, such as extended-spectrum β-lactamase (ESBL)-producing *Enterobacteriaceae* ^2, 10–12^, multidrug-resistant (MDR) *Acinetobacter baumannii* ^13, 14^ and MDR *Enterococcus* spp^15^ have also been demonstrated in veterinary settings.

The first reports of MRSA outbreaks in equine veterinary settings were published in 1997, in a study from Japan which reported the occurrence of a distinct MRSA strain isolated from equine cases of metritis with a putative epidemiological relationship^16^. Subsequent studies have shown that the genotypic characteristics of most MRSA isolates found in the equine populations in Europe, unlike those found in small companion animals, generally differ from the common human clones^4, 17, 18^. However, the majority of the North American MRSA isolates from horses and veterinary staff belong to clonal complex (CC) 8 and have been identified as Canadian epidemic MRSA-5, equivalent to “USA500” or sequence type (ST)8 by multilocus sequence typing (MLST)^6, 19^. Canadian epidemic MRSA-5 (USA-500) has been frequently reported in horses in Canada, but seems to be uncommon among human infections in this country and also restricted to a small number of sites when present^20, 21^. Although MRSA ST8 was also reported in horses from Ireland^22^ and Germany^23^, in Europe MRSA isolates from nosocomial infections in horses are mostly ST254 (a double locus variant of ST8) which also belongs to CC8^5, 24^. However, the livestock-associated (LA) MRSA CC398 has recently emerged in horses, being associated with clinical infections and hospital outbreaks in mainland Europe^17^, including Austria^5–25^, Belgium^26^, Netherlands^6^, Sweden^7^ and Switzerland^27^. Although emerging in mainland Europe, there is only one report of MRSA CC398 in horses in the United Kingdom from 2009 when two horses (one of them without history of travelling outside UK), were found to be positive for MRSA CC398 t011 SCC*mec*IVa^28^.

Biosecurity and infection control are extremely important for equine veterinary settings and some hospitals have implemented rigorous programs. However, active environmental bacteriological surveillance as part of the infection prevention and control strategies for veterinary hospitals are rarely implemented due to concerns over costs and benefits. With the exception of a few studies which investigated the occurrence of MRSA infections in horses or equine hospitals over time^29–32^, long term studies (greater than three years) monitoring environmental MRSA in veterinary hospitals are lacking. In this study, we focus on the value of sustained monitoring of MRSA occurrence in the hospital environment during a five-year active environmental surveillance program designed to monitor the presence of MDR bacteria in an equine veterinary hospital in the United Kingdom. We also describe the distribution and molecular epidemiology of MRSA CC398 isolates circulating in the hospital environment during this period of time which was identified through this surveillance program.

## Results

### Samples processed and MRSA prevalence

Eight hundred and twenty-nine samples were collected from the equine hospital between January 2011 and May 2016, with 540 samples from environmental sites, 208 from SSIs and 81 from hand-plates (Table 1). Overall, MRSA was identified in 62/540 (11.5%) of environmental samples, 6/81 of the hand-plates samples (7.4%) and 13/208 of the SSIs (6.3%). Associated with the cluster of cases and additional environmental swabbing, there were higher numbers of MRSA isolates obtained in 2016 (n = 55) with the majority of isolates obtained from the environment (n = 42), whilst a smaller number was obtained from the SSIs (n = 6) and hand-plates (n = 6). Selected non-duplicate isolates obtained during Jan 2011–May 2016 and identified as CC398 by PCR [n = 38, where 27 were environmental and 11 from SSIs (all 11 obtained from different horses)] were used for further phenotypic and molecular characterisation. Following the SSI cases at the beginning of 2016, environmental follow-up swabbing was performed according to the infection control program. Ninety eight environmental samples and 15 SSIs were collected from June 2016–February 2017; three of the environmental samples (one from an anaesthetics machine Y-piece and two from stables) and two SSIs were MRSA positive. However, all 5 of these MRSA isolates were CC398 negative by PCR.

**Table 1:** Distribution of MRSA positive samples (including CC398) in an equine hospital during the surveillance period 2011–2016.

	2011		2012		2013		2014		2015		2016	
	no.	MRSA positive (CC398)	no.	MRSA positive (CC398)	no.	MRSA positive (CC398)	no.	MRSA positive (CC398)	no.	MRSA positive (CC398)	no.	MRSA Positive (CC398)
ENV samples	112	5 (2) [4.5(1.8)]^*^	101	2 (0) [2.0(0.0)]	102	8 (2) [7.8(2.0)]	37	2 (1) [5.4(2.7)]	57	3 (2) [5.3(3.5)]	131	42 (42) [32.1(32.1)]
Surgical site infections	5	1 (1)	8	0	8	0	55	1 (1)	112	4 (3)	20	7 (7)
Hand plates	34	0	0	—	0	—	0	—	0	—	47	6(4)
Total	151	6 (3)	109	2 (0)	110	8 (2)	92	3 (2)	169	7 (5)	198	55 (53)

All MRSACC398 isolates obtained from 2011–2015 were included in the study; for isolates obtained in 2016 (January–June), in addition to this approach, two and four isolates obtained at different time points were included for sites sampled more than three times (one stable) and six times (one stable) respectively. The SSI surveillance sample size (one isolate/case) was constant throughout the study period.

ENV: environmental.

*Percentage of MRSA and CC398 obtained in each year are shown in the square brackets.

Abbreviations: ENV, environmental.

### Susceptibility testing, detection of virulence and resistance genes

All MRSA isolates characterised here (n = 38) carried *mecA* and also showed resistance to gentamicin (36/38 were resistant and carried the *aacA-aphD* gene), but were not resistant to other aminoglycosides; all the isolates were susceptible to amikacin and only one isolate was resistant to neomycin. In addition, all the isolates were resistant to tetracycline (36/38 carried *tetM*, whilst the remaining 2/38 isolates were positive for *tetK*). Only one isolate was phenotypically resistant to erythromycin and *erm* genes encoding for inducible resistance to clindamycin were rare (*ermA* 3/38, *ermB* 0/38, *ermC* 2/38). Resistance to trimethoprim-sulfamethoxazole was only present in three isolates. Biofilm associated *icaA* and/or *icaD* genes were present in 14/38 and 37/38 isolates, respectively. All isolates were found to lack the *lukS-PV* and *lukF-PV* genes encoding Panton-Valentine leukocidin and the biofilm associated *bap* gene (Table 2).

**Table 2:** Summary of the molecular characterisation of the representative MRSA CC398 isolates from an equine hospital.

Strain	Year	Site (Location)	CC398	SCC*mec* type	*spa*-type	*spa* CC	Resistance phenotype*	Antimicrobial resistance genes
M 1	2011	SSI	+	IVa	t011	*spa* CC011/3423	Gen, Tet	*mecA*,*icaD*,*tetM*,*aacA-aphD*
M 2	2011	ENV (Stable floor)	+	IVa	t011	*spa* CC011/3423	Gen, Tet	*mecA*,*icaD*, *tetM*,*aacA-aphD*
M 3	2011	ENV (Stable floor)	+	IVa	t011	*spa* CC011/3423	Gen, Tet	*mecA*,*icaD*, *tetM*,*aacA-aphD*
M 4	2013	ENV (Stable floor)	+	IVa	t073	Singleton	Enr, Tet	*mecA*,*icaA*, *icaD*, *tetK*, *ermC*
M 5	2013	ENV (Stable floor)	+	IVa	t011	*spa* CC011/3423	Gen, Tet	*mecA*,*icaD*, *tetM*,*aacA-aphD*
M 6	2013	ENV (Stable floor)	+	UT	t011	*spa* CC011/3423	Gen, Tet	*mecA*,*icaD*, *tetM*,*aacA-aphD*
M 7	2014	SSI	+	IVa	t011	*spa* CC011/3423	Gen, Tet	*mecA*,*icaD*, *tetM*,*aacA-aphD*
M 8	2014	ENV (Staff Keyboard)	+	IVa	t786	Singleton	Tet	*mecA*,*icaD*, *tetK*, *ermC*
M 9	2015	ENV (Y-piece no 1)	+	IVd	t064	Singleton	Gen, Sxt, Tet	*mecA*,*icaA*, *icaD*, *tetM*, *aacA-aphD*
M 10	2015	ENV (Stable floor)	+	UT	t064	Singleton	Gen, Sxt, Tet	*mecA*,*icaD*, *tetM*,*aacA-aphD*
M 11	2015	SSI	+	IVd	t064	Singleton	Gen, Sxt, Tet	*mecA*,*icaD*, *tetM*,*aacA-aphD*
M 12	2015	SSI	+	IVa	t588	*spa* CC011/3423	Gen, Enr, Tet	*mecA*,*icaD*, *tetM*,*aacA-aphD*
M 13	2015	SSI	+	IVa	t011	*spa* CC011/3423	Gen, Enr, Tet, Neo	*mecA*,*icaD*, *tetM*,*aacA-aphD*
M 14	2016	SSI	+	IVa	t588	*spa* CC011/3423	Gen, Enr, Tet	*mecA*,*icaD*, *tetM*,*aacA-aphD*
M 15	2016	SSI	+	IVa	t3423	*spa* CC011/3423	Gen, Tet	*mecA*,*icaD*, *tetM*,*aacA-aphD*
M 16	2016	SSI	+	IVa	t011	*spa* CC011/3423	Gen, Tet	*mecA*,*icaA*, *icaD*, *tetM*, *aacA-aphD*
M 17	2016	SSI	+	IVa	t011	*spa* CC011/3423	Gen, Tet	*mecA*,*icaA*, *icaD*, *tetM*, *aacA-aphD*
M 18	2016	SSI	+	IVa	t011	*spa* CC011/3423	Gen, Tet	*mecA*,*icaA*, *icaD*, *tetM*, *aacA-aphD*
M 19	2016	SSI	+	IVa	t011	*spa* CC011/3423	Gen, Tet	*mecA*,*icaA*, *icaD*, *tetM*, *aacA-aphD*
M 20	2016	ENV (Stable floor)	+	IVa	t588	*spa* CC011/3423	Gen, Ery, Tet	*mecA*,*icaA*, *icaD*, *tetM*, *aacA-aphD*
M 21	2016	ENV (Stable floor)	+	IVa	t011	*spa* CC011/3423	Gen, Tet	*mecA*,*icaD*, *tetM*,*aacA-aphD*
M 22	2016	ENV (Stable floor)	+	IVa	t011	*spa* CC011/3423	Gen, Tet	*mecA*,*icaD*, *tetM*,*aacA-aphD*
M 23	2016	ENV (Stable floor)	+	IVa	t3423	*spa* CC011/3423	Gen, Tet	*mecA*,*icaD*, *tetM*,*aacA-aphD*
M 24	2016	ENV (Stable floor)	+	IVa	t588	*spa* CC011/3423	Gen, Enr, Tet	*mecA*,*icaD*, *tetM*,*aacA-aphD*
M 25	2016	ENV (Stable drain)	+	IVa	t011	*spa* CC011/3423	Gen, Tet	*mecA*,*icaD*, *tetM*,*aacA-aphD*
M 26	2016	ENV (Stable floor)	+	IVa	t011	*spa* CC011/3423	Gen, Tet	*mecA*,*icaD*, *tetM*,*aacA-aphD*
M 27	2016	ENV (Stable wall)	+	IVa	t011	*spa* CC011/3423	Gen, Tet	*mecA*,*icaD*, *tetM*,*aacA-aphD*
M 28	2016	ENV (Stable brush)	+	IVa	t011	*spa* CC011/3423	Gen, Tet	*mecA*,*icaD*, *tetM*,*aacA-aphD*
M 29	2016	ENV (Stable floor)	+	IVa	t011	*spa* CC011/3423	Gen, Tet	*mecA*,*icaA*, *icaD*, *tetM*, *aacA-aphD*
M 30	2016	ENV (Stable floor)	+	IVa	t011	*spa* CC011/3423	Gen, Tet	*mecA*,*icaA*, *icaD*, *tetM*, *aacA-aphD*
M 31	2016	ENV (ICU Keyboard)	+	IVa	t011	*spa* CC011/3423	Gen, Tet	*mecA*,*icaA*, *icaD*, *tetM*, *aacA-aphD*
M 32	2016	ENV (Y-piece)	+	IVa	t011	*spa* CC011/3423	Gen, Tet	*mecA*,*icaD*, *tetM*,*aacA-aphD*
M 33	2016	ENV (Reception keyboard)	+	IVa	t1985	*spa* CC011/3423	Gen, Tet	*mecA*,*icaD*, *tetM*,*aacA-aphD*
M 34	2016	ENV (Student keyboard)	+	UT	t011	*spa* CC011/3423	Gen, Tet	*mecA*,*tetM*,*aacA-aphD*
M 35	2016	ENV (Hand plate)	+	IVa	t1985	*spa* CC011/3423	Gen, Tet	*mecA*,*icaA*, *icaD*, *tetM*, *ermA*,*aacA-aphD*
M 36	2016	ENV (Hand plate)	+	IVa	t011	*spa* CC011/3423	Gen, Tet	*mecA*,*icaA*, *icaD*, *tetM*, *aacA-aphD*
M 37	2016	ENV (Hand plate)	+	IVa	t011	*spa* CC011/3423	Gen, Tet	*mecA*,*icaA*, *icaD*, *tetM*, *ermA*,*aacA-aphD*
M 38	2016	ENV (Hand plate)	+	IVa	t011	*spa* CC011/3423	Gen, Tet	*mecA*,*icaA*, *icaD*, *tetM*, *ermA*,*aacA-aphD*

*Resistance phenotype shown to non β-lactams antimicrobials only.

Abbreviations: Enr, Enrofloxacin; Gen, gentamicin; Neo, neomycin; SXT, trimethoprim/sulfamethoxazole; Tet, tetracycline; ENV, Environment; SSI, surgical site infection; UT, Un-typeable.

### Molecular characterisation

MLST typing of six selected clinical and environmental isolates obtained from early 2016 (four from SSIs, one from a stable and one from a computer keyboard) identified them all as ST398. Retrospective molecular typing was performed with the CC398-specific PCR assay on all 81 MRSA isolates obtained from the active surveillance program (Jan 2011–May 2016), which identified that 65/81 (80.3%) MRSA isolates obtained in the past 5 years belonged to CC398. Among the isolates from 2016, all except two MRSA isolates which resulted from hand-plate sampling performed during the study period, belonged to CC398 (n = 53/55), whilst this lineage had a lower frequency amongst the isolates from the previous years (2011, n = 3/6; 2013, n = 2/8; 2014, n = 2/3; 2015, n = 5/7) and was not isolated in 2012.

SCC*mec* typing of selected MRSA CC398 isolates obtained from 2011 to 2016 (n = 38), identified 33/38 CC398 MRSA isolates obtained throughout all years as type IVa and 2/38 (both obtained in 2015) as type IVd. Three isolates obtained from 2013, 2015 and 2016 were un-typeable by this method.

*spa* typing identified seven different *spa* types in the CC398 MRSA isolates with t011 being the most frequent (24/38), followed by t588 (4/38), t064 (3/38), t1985 (2/38), t3432 (2/38), t073 (1/38) and t786 (1/38). Taken together, the MLST, SCC*mec* and *spa* typing showed that the most prevalent MRSA type present from 2011 to 2016 in the hospital environment was CC398-IVa-t011 which was identified in 24/38 (63%) of the MRSA CC398 isolates.

**Figure 1:**
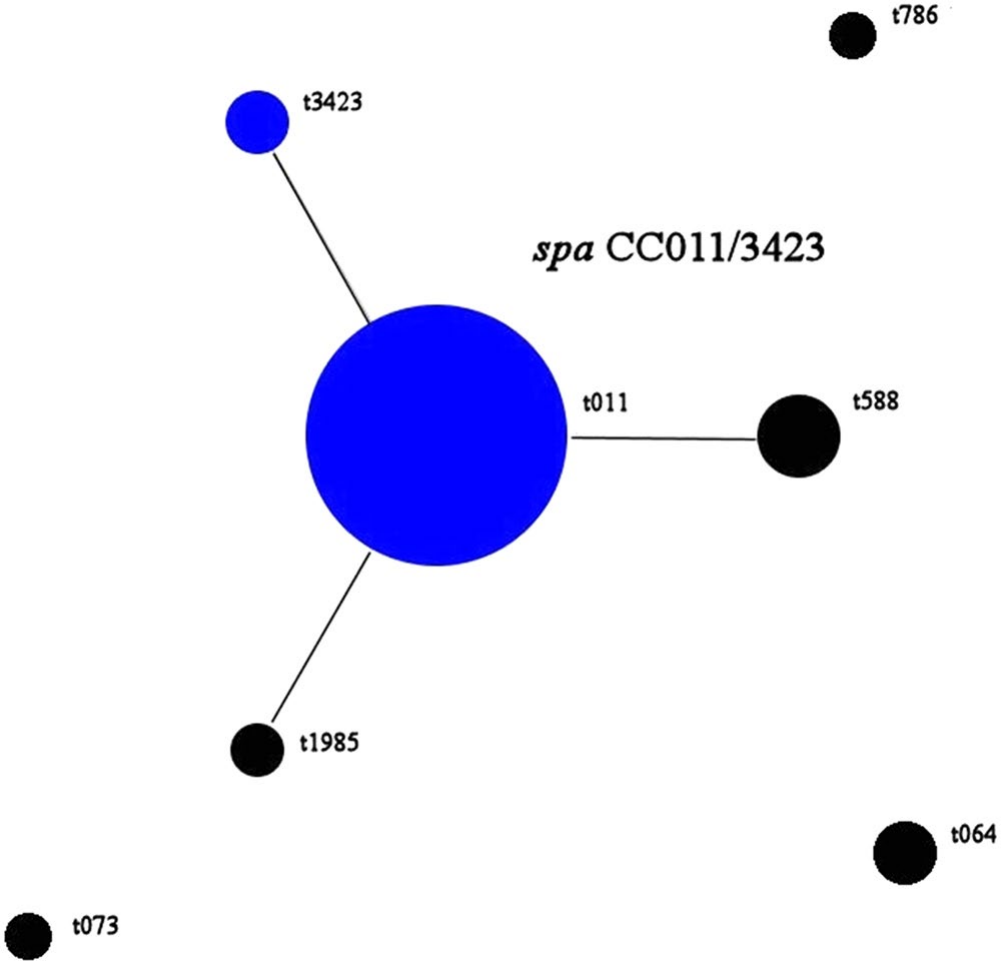
Population snapshot of MRSA CC398 isolates (2011–2016) according to Based Upon Repeat Pattern (BURP) analysis results. The diameter of a dot is proportional to the number of isolates of the corresponding *spa* type. Blue dots represent group founders as defined in the Methods section.

Using the default analysis parameters of BURP, the *spa* types identified in this study clustered into one main *spa* CC (*spa* CC011/3423) with two designated group founders and 3 singletons [t073, t786, t064 (Table 3 and Fig. 1)]. In *spa* CC011/3423 *spa* types t011 and t3423 had identical founder scores.

**Table 3:** MRSA *spa* types and repeat profiles according to the results of Based Upon Repeat Pattern (BURP) cluster analysis.

*spa* type	Repeat profile	No. of isolates	*spa* CCs
t011	r08-r16-r02-r25-r34-r24-r25	25	*spa* CC011/3423
t588	r08-r16-r02- r24-r25	4	*spa* CC011/3423
t1985	r08-r16-r02-r25-r34	2	*spa* CC011/3423
t3432	r08-r16-r02-r25-r34-r24	2	*spa* CC011/3423
t073	r08-r16-r02-r16-r13-r17-r34-r16-r34	1	Singleton
t786	r07-r12-r21-r17-r13-r34-r34-r33-r34	1	Singleton
t064	r11-r19-r12-r05-r17-r34-r24-r34-r22-r25	3	Singleton

### Hospital epidemiology of MRSA CC398

MRSA CC398-IVa*-*t011 was the most prevalent type identified in this study and was identified in the hospital environment and in SSIs, as well as on hand-plates in 2016. Retrospective analysis of archived isolates showed that the first isolation of this genotype was in 2011 when it was obtained from both the environment (stable floor) and from one case that developed an SSI, a pattern that was also seen in 2013. A single isolation of *spa* type t073 from a stable floor was also found in 2013. During environmental surveillance in 2014, two different CC398 *spa* types were identified, where t011 was found in an SSI, whilst t786 was identified on a computer keyboard. Interestingly, in 2015 a cluster of CC398-IVd-t064 isolates was observed, where an initial isolate was obtained from the anesthetic machine y-piece, followed by the isolation from a stable (after three weeks) and an SSI four months later; this *spa* type seems to have been quickly eliminated from the environment and did not recur during environmental sampling. At the same time, t011 was isolated only once from a SSI infection during 2015 environmental monitoring.

The cluster of six MRSA SSIs in 2016 prompted increased environmental sampling and subsequently increased isolation from the environment. Molecular typing of the MRSA isolates obtained in 2016 identified introduction of four different *spa* types between February–June 2016 (t011, t588, t3423 and t1985) the first three of which were associated with SSIs indicating possible new introductions into the hospital.

Of the six MRSA positive hand-plates identified during this cluster of cases in 2016, four isolates were MRSA CC398, of which three were *spa* type t011 (one from a nurse and two from students). Another hand plate isolate (from a clinical staff member) was typed as t1985 and was also obtained from the reception keyboard two months earlier, but was not isolated from any SSIs. Overall, *spa* type t011 was the most prevalent type isolated from the environment (stable floors, stable cleaning brushes, ICU keyboard, student common room keyboard), as well as from the SSIs and hand-plates.

## Discussion

This is the first long-term study investigating hospital epidemiology of MRSA in equine veterinary settings. Previous studies have shown the potential of MRSA transmission between owners and pets, or veterinary staff and patients, highlighting the zoonotic risks associated with this pathogen^21, 33^. Although MRSA CC398 or LA-MRSA is common amongst farmers (particularly pig farmers) in northern Europe, originally it was considered an unusual human coloniser. However, recent studies indicate that human infections with this lineage may be increasing in countries like Denmark where the incidence of MRSA CC398-IIa infection in people (with or without livestock exposure) showed an annual increase of 66% from 2004 to 2011, although this is only represented by 151 clinical cases during this period^34, 35^.

Our study showed that MRSA was present in several areas of the equine hospital environment and was associated with a SSI in every year of this study. The prevalence of MRSA detected from the environmental during the first four years of the active surveillance program of (2011–2015) was 4% which is similar to a previous study performed during a non-outbreak period^36^, but lower than the prevalence found by Van Balen *et al*.^31^ during a one year long surveillance study (8.6%). Our study also found 6.3% of SSIs to be associated with MRSA infection whilst a large study in Germany found MRSA to be associated with 9.4% of wound infections in horses and with the majority of isolates typed to CC398^37^. However, during 2016, a cluster of SSIs cases prompted increased environmental surveillance which identified increased MRSA contamination and indicated a persistence of MRSA in this UK veterinary hospital; these data triggered enhanced infection control protocols including a deep clean of all the intensive care stables which was successful in removing the MRSA. Staff at all levels were involved in the deep clean action (nurses, clinicians, senior management staff) which is in line with findings from another veterinary hospital study which suggests that interventions based on a general consensus amongst hospital management staff, infection control teams and clinicians are required in order to successfully remove MRSA from the hospital environment^30^.

Molecular characterisation of the isolates obtained during this five-year period (Jan 2011–May 2016) typed 80% of the MRSA isolates to CC398 and identified the emergence of LA-MRSA CC398, in particular ST398 in horses in the UK. Moreover, *spa* typing of isolates also revealed a variety of *spa* types suggesting multiple introductions of MRSA CC398 over the five-year surveillance period. This finding is also supported by the BURP analysis which identified a main *spa* CC (*spa* CC011/3423) which appears to include closely related isolates and three singletons t064, t073 and t786 which seem to represent new introductions. Data analysis shows that the main *spa* CC (*spa* CC011/3423) was isolated throughout 2011–2016 whilst new introductions occurred in 2013 (MRSA CC398-t073-IVa obtained from a stable floor), 2014 (MRSA CC398-t786-IVa isolated from staff keyboard) and 2015 (MRSA CC398-t064-IVd/UT, found in a SSI and in two environmental samples). In addition, BURP analysis showed that *spa* CC011/3423 had two main group founders, of which t011 was isolated throughout 2011–2016, whilst the second group founder t3423 (as well as the other group members, t588 and t1985) were isolated from the end of 2015 onwards suggesting microevolution of t011. BURP analysis indicates that mutational events (mainly deletions) are likely to have occurred in this period of time which resulted in *spa* type diversification. Interestingly, MRSA CC398-IVa*-*t011 was the most prevalent clone identified in this study with 63.2% of MRSA CC398 isolates typed to this clone. This is in line with findings from a recent survey conducted in Denmark, where 59% of the MRSA isolates from horses were typed as CC398-IVa*-*t011, further confirming the emergence of this strain in the European equine population^38^. Recent studies have shown that certain *spa* types (including t011) and SCC*mec* types (such as IV and V) are significantly associated with particular phylogenetic clades and that within the MRSA CC398 lineage, a sub-lineage has emerged which is designated as the “equine clade C” and which showed an association with colonisation and infection in horses and equine hospital personnel^39^. In addition to resistance to tetracycline (a characteristic of MRSA CC398), the equine clade C isolates also exhibit *aacA*-*aphD* encoded gentamicin resistance^5^ which had been identified in our isolates and which supports their typing to the “equine clinic clade”.

Data presented in this study demonstrated a pattern of MRSA environmental contamination suggesting introduction or reintroduction of various MRSA strains and maintenance of strains over time in the environment, with some strains more persistent than others. In this study, we showed that certain MRSA CC398 *spa* types were only occasionally identified in the environment or SSIs (i.e. t073), whilst MRSA CC398-IVa*-*t011 was repeatedly isolated during the five-year study period. Whether this was the result of persistence in the environment or constant re-introduction, possibly due to a high prevalence of this clone in the equine population, or due to introduction through new staff or students transiently colonised with this clone, needs to be further investigated. Further studies using whole genome sequencing (WGS) are planned for the CC398 and non-CC398 MRSA isolates collected during the study period to map the introduction, persistence and spread of this lineage through the hospital environment. In addition, WGS will provide a better understanding of MRSA evolution within the UK and European equine populations.

The high prevalence of biofilm-related genes *icaA* and *icaD* (36.8% and 100% respectively) in the CC398 isolates may explain their likely persistence in the environment due to the production of biofilms protecting micro-organisms from disinfectant action which may have contributed to failure of decontamination prior to the reuse of stables. It has been previously shown that routine cleaning procedures do not reliably remove biofilms from surfaces, and this may explain the unexpected failure of decontamination encountered during outbreak episodes despite adherence to infection control guidelines^40^.

Identification of the main circulating MRSA-CC398-IVa-t011 on hand-plates from students and staff and work surfaces further emphasises the role that hand-hygiene may play in the transmission of MRSA in veterinary hospitals. It is important to emphasise the importance of biosecurity and hand-hygiene practices by veterinary hospital personnel, primarily their compliance with hand washing which can be often overlooked in veterinary hospitals^41^. In addition, findings from surveillance studies such as this are a reminder of the importance of compliance with biosecurity protocols and procedures when wearing gloves and especially, the need to change gloves at critical points during interventions. A recent Swedish study showed that one of the most common barriers to compliance with hand-hygiene in equine hospitals is an insufficient supply of hand sanitiser or lack of accessible places to wash hands, all which can be easily resolved when managers are supportive of the infection control team^29^.

In this study, both MRSA CC398 *spa* type t011 and t064 have been isolated from the anaesthetic machine y-piece, which is difficult to clean and disinfect and is included in the routine screening due to its role as a possible source of cross-contamination between horses. This study demonstrated that incorporating high risk areas (such as intensive care units) or equipment (anaesthetic machine y-piece) in routine environmental bacterial monitoring, allows quick intervention and implementation of enhanced cleaning and disinfection, leading to elimination of likely reservoirs for veterinary hospital acquired infections.

The cluster of cases in 2016 and especially the identification of three different *spa* types associated with a small number of SSI, suggests that horses can be MRSA carriers and may have played a role in the introduction and movement of this bacterium within the hospital. In addition, the long-term hospital contamination and high prevalence of MRSA CC398 identified in this study may also suggest a high prevalence of this clone in the UK equine population and further work is needed to ascertain this. Here, we have used a PCR based method to rapidly identify MRSA strains belonging to CC398 and this provides a useful diagnostic tool for surveillance of colonisation/infection with this pathogen in equine clinics.

In conclusion, this study has revealed multiple introductions of MRSA in a UK equine hospital and, most importantly, the persistence and spread of CC398, an emerging zoonotic pathogen only sporadically recorded in the UK so far. The study also emphasizes the importance of sustained active environmental surveillance which allowed detection and monitoring of MRSA occurrence, as well as the implementation of infection control policies designed to protect equine patients, the hospital staff and the wider public from exposure to this important zoonotic pathogen.

## Methods

### Active surveillance as part of a hospital infection control program

In January 2011 an environmental monitoring scheme was piloted for six months in a large Equine Referral Hospital (receiving 1800 patients a year), to obtain baseline reference data for a long-term active surveillance program. The pilot study identified a number of hospital surfaces that can occasionally become contaminated with potential nosocomial pathogens and could play a role in transmission. Subsequently MRSA, MDR (resistant to three or more drug classes) *Enterococcus*, MDR and/or ESBL *Enterobacteriaceae* and non-fermentative Gram negative organisms (*Pseudomonas* spp., *Acinetobacter* spp.), and *Salmonella* spp. were identified as target pathogens for routine active environmental surveillance. Surfaces from high risk areas such as surgical theatres, intensive care units, treatment areas, recovery boxes, equipment (endotracheal tubes, anesthetic equipment), as well as human high contact areas (computer keyboards, door handles, phone handles) were included in the subsequent routine surveillance on a rotating basis. Surgical site infections (SSIs) were also included in the surveillance activity to monitor the involvement of nosocomial pathogens in these infections.

The frequency of sampling was intended to be monthly, but ultimately this was determined by the hospital infection control team according to clinical load and the occurrence of clinical infection with potential nosocomial pathogens. Although active environmental surveillance included screening for target pathogens (MRSA, MDR *Enterococcus*, MDR and/or ESBL Gram negative bacteria and *Salmonella* spp.), in this paper we focus on the long-term surveillance of MRSA in this equine hospital. This was a retrospective study and owner consent was obtained on admission of horses via generic consent which allows for diagnostic samples to be used for research. The environmental and hand-hygiene sampling protocols were approved by the Infection Control and Biosecurity Committee at the Institute of Veterinary Science when it was first implemented in 2011. Occasional hand-plate sampling (aiming to staff reinforce hand-hygiene) was included as part of the Hospital Infection Control policy and the Committee agreed that this would be voluntary and would not require informed consent from participants. All methods were carried out in accordance with specific guidelines and regulations including relevant positive and quality controls.

### MRSA isolates

Infection control protocols introduced as part of this program, recommended implementation of mechanical cleaning and disinfection of MRSA positive areas followed by repeated swabbing until samples were culture negative; this approach led to multiple isolates obtained from the same area, especially in 2016. For the 2011–2015 surveillance periods, only one MRSA isolate obtained from each site (i.e., stables floor, stable brush, anesthetic machine y-piece, computer key-boards) was included for phenotypic and molecular testing. However, for 2016 if one area (i.e., stable) was swabbed more than three times, two isolates obtained at two different time points were included, or if the same area was swabbed more than six times, four isolates were included for molecular testing. For SSIs, only one isolate per one case was included with this approach being consistent throughout the study period.

### Hand-plate sampling

The role of hand hygiene in the transmission of hospital acquired infection was monitored and hand-plate sampling was performed occasionally as part of the infection control program. This process was anonymous as a code was given to each plate to identify the sampled category (staff, students, clinical, non-clinical area) and not individuals. Hand plating was performed randomly without prior notice during the mid-morning of a routine work day. Staff members did not sanitise their hands prior to sampling (unless by chance) in order to capture transient bacterial flora on hands during their routine activity.

### Samples, processing and antimicrobial susceptibility testing

Sterile pre-moistened electrostatic Swiffer^®^ wipes (Procter & Gamble, Ohio, US) were used to sample various surfaces, using sterile gloves which were changed between samples. Sample collection was performed by a trained technician who used one side of the electrostatic wipes to sample a representative surface size (approximatively 0.5 m^2^), then the cloth was folded and placed in bottles containing 250 ml of buffered peptone water (BPW). Samples were transported to the on-site laboratory immediately after collection and incubated at 37 °C ± 2 for 24 hours. For MRSA isolation, 10 µl of enriched BPW were plated directly onto a *Brilliance™* MRSA 2 Agar plate and a 500 µl BPW aliquot was added to 7.5% NaCl Nutrient Broth with overnight incubation at 37 °C ± 2. After the enrichment step in the salt broth, a second *Brilliance™* MRSA 2 Agar plate was inoculated as for primary cultures. Swabs collected aseptically from discharging SSIs were screened for MRSA using the same culture protocol as for environmental samples. Presumptive MRSA isolates obtained from environmental samples and SSIs were subcultured onto 5% sheep blood agar (SBA, all media from Oxoid, Basingstoke, UK) for further testing.

Ad hoc hand plate sampling was performed for hospital staff (clinicians, nurses, yard staff and receptionists) and students twice (2011 and 2016) during the five-year surveillance period. Samples were collected by impression of fingers and thumb of the dominant hand on a SBA plate and incubated for 24 hours at 37 °C ± 2. Colonies with morphological characteristics suggestive of *S*. *aureus* were sub cultured onto *Brilliance™* MRSA 2 Agar and investigated further only if typical growth was present after 24 hours of incubation at 37 °C ± 2.

Antimicrobial susceptibility testing was performed by the disk-diffusion method according to the European Committee on Susceptibility Testing (EUCAST) guidelines^42^. Antimicrobial testing was performed with an extended panel for environmental and SSI isolates including amikacin, ampicillin, cefoxitin, ceftiofur, enrofloxacin, erythromycin, gentamicin, oxacillin, oxytetracycline, penicillin, trimethoprim-sulfamethoxazole (all media and discs from Oxoid, Basingstoke, UK). Interpretation of susceptibility results was performed according to EUCAST or the Clinical and Laboratory Standards Institute (CLSI)^43^ for veterinary specific antimicrobials (i.e., ceftiofur, enrofloxacin). *S*. *aureus* (ATCC 25923) was used as quality control for susceptibility testing.

### Confirmation of MRSA and molecular characterisation of isolates

Presumptive MRSA colonies grown overnight on SBA were used for biochemical identification using gram-positive identification plates (GPID) (TREK Diagnostic Systems Ltd., Cleveland, OH, USA) and to prepare cell lysates for DNA extraction by heating a suspension of cells at 100 °C for 10 minutes. A multiplex PCR assay targeting the *femA* ^44^, *nucA* and *mecA* ^45^ genes was used for confirmation of MRSA status.

Molecular typing was performed on a selection of isolates (n = 6) by MLST as previously described^46^. Confirmation of sequence type (ST) 398 by MLST in the isolates, prompted retrospective screening of all the MRSA isolates obtained from the environment (n = 62), SSI (n = 13) and hand plates (n = 6) in the past five years with a CC398-specific PCR^47^. Non-duplicate isolates identified as CC398 were further characterised by staphylococcal chromosomal cassette *mec* (SCC*mec*) and *spa* gene typing. SCC*mec* typing (type I to type V) was performed according to Zhang *et al*.^48^. *S*. *aureus* protein A (*spa*) typing was performed as previously described^49^. Amplification was followed by Sanger sequencing to identify sequence variation of the polymorphic region X of the *spa* gene and *spa* types were determined using the *spa*Typer software (http://spatyper.fortinbras.us).

Based Upon Repeat Pattern (BURP) was used to determine clonal relatedness from *spa* repeat regions and cluster *spa* types (*spa* CCs) of isolates by using Ridom StaphType (version 2.2.1) software (Ridom GmbH, Würzburg, Germany). The default parameters for BURP analysis were applied as previously described^50^. Interpretation of BURP clusters was performed according to Mellman *et al*.^51^ where a group founder in clusters of at least three different *spa* types, is described as the *spa* type with the highest founder score.

All PCR-confirmed CC398 MRSA isolates were screened for the presence of virulence genes *lukS-PV*, *lukF-PV* which encode the Panton-Valentine leukocidin^52^, for genes associated with biofilm production (*bap*, *icaA*, *icaD*)^53^ and antimicrobial resistance genes (*ermA*, *ermB*, *ermC*, *tetK*, *tetM*, *aacA-aphD*)^54, 55^. Positive and negative controls for PCR reactions were included in each assay.

### MRSA follow-up samples and environmental cleaning

Isolation of MRSA from SSI and environmental samples at the beginning of 2016 was followed by a deep clean action. This involved closure of the affected stable blocks and a thorough clean involving removal of all items from the stable, scrubbing to remove organic matter using an alkaline detergent and warm water, pressure washing or steam cleaning, allowing the stable to dry, followed by spraying of Virkon S (1–2% solution) onto walls and floor and allowing it to dry for a minimum of 24 hours. All associated passageways, outside areas and drains were fully cleaned and any necessary maintenance performed – floors and walls painting, rubber replacement/sealing etc. Horses were not reintroduced into the stables until re-swabbed samples were MRSA negative.

Following the last SSI positive case (May 2016), environmental samples were collected monthly to monitor the presence of MRSA in the environment.

## References

[1] La Force, F. M. The Control Of Infections In Hospitals: 1750 To 1950 in *Prevention And Control Of* Nosocomial *Infections* (ed. Wenzel, R.P.). 1–12 (Williams & Wilkins, 1993).

[2] Wieler LH, Ewers C, Guenther S, Walther B, Lübke-Becker A (2011). Methicillin-resistant staphylococci (MRS) and extended-spectrum beta-lactamases (ESBL)-producing Enterobacteriaceae in companion animals: nosocomial infections as one reason for the rising prevalence of these potential zoonotic pathogens in clinical samples. Int J Med Microbiol.

[3] Seguin JC (1999). Methicillin-resistant *Staphylococcus aureus* outbreak in a veterinary teaching hospital: potential human-to-animal transmission. J Clin Microbiol.

[4] Cuny C (2006). Emergence of MRSA infections in horses in a veterinary hospital: strain characterisation and comparison with MRSA from humans. Euro Surveill.

[5] Cuny C, Strommenger B, Witte W, Stanek C (2008). Clusters of infections in horses with MRSA ST1, ST254, and ST398 in a veterinary hospital. Microb Drug Resist.

[6] van Duijkeren E (2010). Methicillin-resistant *Staphylococcus aureus* in horses and horse personnel: an investigation of several outbreaks. Vet Microbiol.

[7] Bergström K, Aspan A, Landén A, Johnston C, Grönlund-Andersson U (2012). The first nosocomial outbreak of methicillin-resistant *Staphylococcus aureus* in horses in Sweden. Acta Vet Scand.

[8] Schwaber MJ (2013). Clonal transmission of a rare methicillin-resistant *Staphylococcus aureus* genotype between horses and staff at a veterinary teaching hospital. Vet Microbiol.

[9] Kuroda T (2016). Meticillin-resistant *Staphylococcus aureus* colonisation and infection in Thoroughbred racehorses and veterinarians in Japan. Vet Rec.

[10] Sanchez S (2002). Characterization of multidrug-resistant *Escherichia coli* isolates associated with nosocomial infections in dogs. J Clin Microbiol.

[11] Walther B (2013). Suspected nosocomial infections with multi-drug resistant *E*. *coli*, including extended-spectrum beta-lactamase (ESBL)-producing strains, in an equine clinic. Berl Munch Tierarztl.

[12] Timofte D, Maciuca IE, Williams NJ, Wattret A, Schmidt V (2016). Veterinary Hospital Dissemination of CTX-M-15 Extended-Spectrum Beta-Lactamase–Producing *Escherichia coli* ST410 in the United Kingdom. Microb Drug Resist.

[13] Endimiani A (2011). *Acinetobacter baumannii* isolates from pets and horses in Switzerland: molecular characterization and clinical data. J Antimicrob Chemoth.

[14] Müller S, Janssen T, Wieler LH (2013). Multidrug resistant *Acinetobacter baumannii* in veterinary medicine–emergence of an underestimated pathogen?. Berl Munch Tierarztl.

[15] Boerlin P, Eugster S, Gaschen F, Straub R, Schawalder P (2001). Transmission of opportunistic pathogens in a veterinary teaching hospital. Vet Microbiol.

[16] Shimizu A (1997). Genetic analysis of equine methicillin-resistant *Staphylococcus aureus* by pulsed-field gel electrophoresis. J Vet Med Sci.

[17] Cuny C (2016). Methicillin-resistant *Staphylococcus aureus* from infections in horses in Germany are frequent colonizers of veterinarians but rare among MRSA from infections in humans. One Health.

[18] Monecke S (2011). A field guide to pandemic, epidemic and sporadic clones of methicillin-resistant *Staphylococcus aureus*. PloS one.

[19] Weese JS (2005). Community-associated methicillin-resistant *Staphylococcus aureus* in horses and humans who work with horses. J Am Vet Med Assoc.

[20] Simor AE (2002). Laboratory characterization of methicillin-resistant *Staphylococcus aureus* in Canadian hospitals: results of 5 years of national surveillance, 1995–1999. J Infect Dis.

[21] Weese J (2005). Methicillin-resistant *Staphylococcus aureus* in Horses and Horse Personnel, 2000–2002. Emerg Infect Dis.

[22] Moodley A (2006). spa typing of methicillin-resistant *Staphylococcus aureus* isolated from domestic animals and veterinary staff in the UK and Ireland. J Antimicrob Chemoth.

[23] Walther B (2009). Comparative molecular analysis substantiates zoonotic potential of equine methicillin-resistant *Staphylococcus aureus*. J Clin Microbiol.

[24] Baptiste KE (2005). Methicillin-resistant staphylococci in companion animals. Emerg Infect Dis.

[25] Loncaric I (2014). Identification and characterization of methicillin-resistant *Staphylococcus aureus* (MRSA) from Austrian companion animals and horses. Vet Microbiol.

[26] Hermans K (2008). MRSA clone ST398-SCCmec IV as a cause of infections in an equine clinic. Vlaams Diergen Tijds.

[27] Sieber S (2011). Evolution of multidrug-resistant *Staphylococcus aureus* infections in horses and colonized personnel in an equine clinic between 2005 and 2010. Microb Drug Resist.

[28] Loeffler A (2009). First isolation of MRSA ST398 from UK animals: a new challenge for infection control teams?. J Hosp Infect.

[29] Bergström K, Grönlund U (2014). A pre-and post-intervention study of infection control in equine hospitals in Sweden. Acta Vet Scand.

[30] Bergström K (2012). Infection prevention and control interventions in the first outbreak of methicillin-resistant *Staphylococcus aureus* infections in an equine hospital in Sweden. Acta Vet Scand.

[31] Van Balen J (2014). Molecular epidemiology of environmental MRSA at an equine teaching hospital: introduction, circulation and maintenance. Vet Res.

[32] Bergström K, Bengtsson B, Nyman A, Andersson UG (2013). Longitudinal study of horses for carriage of methicillin-resistant *Staphylococcus aureus* following wound infections. Vet Microbiol.

[33] Nienhoff U (2009). Transmission of methicillin-resistant *Staphylococcus aureus* strains between humans and dogs: two case reports. J Antimicrob Chemoth.

[34] Köck R (2013). Livestock-associated methicillin-resistant *Staphylococcus aureus* (MRSA) as causes of human infection and colonization in Germany. PloS one.

[35] Larsen J (2015). Methicillin-resistant *Staphylococcus aureus* CC398 is an increasing cause of disease in people with no livestock contact in Denmark, 1999 to 2011. Euro Surveill.

[36] Hoet AE (2011). Environmental methicillin-resistant *Staphylococcus aureus* in a veterinary teaching hospital during a nonoutbreak period. Vector Borne Zoonotic Dis.

[37] Vincze S (2014). Alarming proportions of methicillin-resistant *Staphylococcus aureus* (MRSA) in wound samples from companion animals, Germany 2010–2012. PLoS One.

[38] Islam MZ (2017). Horses in Denmark are a reservoir of diverse clones of methicillin-resistant and-susceptible *Staphylococcus aureus*. Front Microbiol.

[39] Abdelbary MM (2014). Phylogenetic analysis of *Staphylococcus aureus* CC398 reveals a sub-lineage epidemiologically associated with infections in horses. PloS one.

[40] Pajkos A, Vickery K, Cossart Y (2004). Is biofilm accumulation on endoscope tubing a contributor to the failure of cleaning and decontamination?. J Hosp Infect.

[41] Gyles C (2009). Infection control in veterinary clinics. Can Vet J.

[42] Matuschek E, Brown D, Kahlmeter G (2014). Development of the EUCAST disk diffusion antimicrobial susceptibility testing method and its implementation in routine microbiology laboratories. Clin Microbiol Infec.

[43] Clinical and Laboratory Standards Institute. Methods for dilution antimicrobial susceptibility tests for bacteria that grow aerobically; Approved Standard-Ninth Edition (M07-A9). *Wayne*, *PA: Clinical and Laboratory Standards Institute* **29**, 25–27 (2012).

[44] Brakstad OG, Aasbakk K, Maeland JA (1992). Detection of *Staphylococcus aureus* by polymerase chain reaction amplification of the *nuc* gene. J Clin Microbiol.

[45] Francois P (2003). Rapid detection of methicillin-resistant *Staphylococcus aureus* directly from sterile or nonsterile clinical samples by a new molecular assay. J Clin Microbiol.

[46] Enright MC (2002). The evolutionary history of methicillin-resistant *Staphylococcus aureus* (MRSA). P Natl Acad Sci Usa.

[47] Stegger M (2011). Rapid PCR detection of *Staphylococcus aureus* clonal complex 398 by targeting the restriction-modification system carrying *sau*1-*hsd*S1. J Clin Microbiol.

[48] Zhang K, McClure J-A, Elsayed S, Louie T, Conly JM (2005). Novel multiplex PCR assay for characterization and concomitant subtyping of staphylococcal cassette chromosome mec types I to V in methicillin-resistant *Staphylococcus aureus*. J Clin Microbiol.

[49] Strommenger B (2008). *spa* typing of *Staphylococcus aureus* as a frontline tool in epidemiological typing. J Clin Microbiol.

[50] Mellmann A (2007). Based Upon Repeat Pattern (BURP): an algorithm to characterize the long-term evolution of *Staphylococcus aureus* populations based on *spa* polymorphisms. BMC microbiol.

[51] Mellmann A (2008). Characterization of clonal relatedness among the natural population of *Staphylococcus aureus* strains by using *spa* sequence typing and the BURP (based upon repeat patterns) algorithm. J Clin Microbiol.

[52] Lina G (1999). Involvement of Panton-Valentine leukocidin—producing *Staphylococcus aureus* in primary skin infections and pneumonia. Clin Infect Dis.

[53] Vancraeynest D, Hermans K, Haesebrouck F (2004). Genotypic and phenotypic screening of high and low virulence *Staphylococcus aureus* isolates from rabbits for biofilm formation and MSCRAMMs. Vet Microbiol.

[54] Strommenger B, Kettlitz C, Werner G, Witte W (2003). Multiplex PCR assay for simultaneous detection of nine clinically relevant antibiotic resistance genes in *Staphylococcus aureus*. J Clin Microbiol.

[55] Sutcliffe J, Grebe T, Tait-Kamradt A, Wondrack L (1996). Detection of erythromycin-resistant determinants by PCR. Antimicrob Agents Chemother.

